# Effects of *Moringa oleifera* Leaf Peptide on Hypoglycemic Activity In Vitro and Postprandial Glycemic Response in Beagle Dogs

**DOI:** 10.3390/ani15162361

**Published:** 2025-08-11

**Authors:** Wencan Wang, Ling Xu, Yong Cao, Guo Liu, Yan Zhang, Xin Mao

**Affiliations:** 1 Chongqing Sweet Pet Products Co., Ltd., Chongqing 400000, China; xuling1114684263@163.com (L.X.); andy.mao@chinasweetpet.com (X.M.); 2 Guangdong Provincial Key Laboratory of Nutraceuticals and Functional Foods, College of Food Science, South China Agricultural University, Guangzhou 510642, China; caoyong2181@scau.edu.cn; 3 College of Light Industry and Food, Zhongkai University of Agriculture and Engineering, Guangzhou 510225, China; liuguo@zhku.edu.cn; 4 Department of Animal Nutrition and Feed, College of Biological Engineering, Sichuan Water Conservancy Vocational College, Chengdu 611200, China; zhangyan09142@163.com

**Keywords:** *Moringa oleifera* leaf peptide, hypoglycemic activity, estimated glycemic index, postprandial glycemic response, functional foods, dogs

## Abstract

*Moringa oleifera* leaf (MOL) and their extracts have been demonstrated to possess hypoglycemic effects in a variety of species, but they are still unknown in dogs. This study examined the effects of *Moringa oleifera* leaf peptide (MOLP) on α-amylase and α-glucosidase activities. Furthermore, we assessed the impact of MOLP on the estimated glycemic index (eGI) of snacks in vitro and the postprandial glycemic response in dogs. The findings indicated that MOLP exhibited significant inhibitory activities against α-amylase (IC_50_ = 2.29 ± 0.10 mg/mL) and α-glucosidase (IC_50_ = 2.80 ± 0.04 mg/mL). Moreover, the MOLP-containing snacks exhibited a lower rate of starch hydrolysis during in vitro digestion, leading to a notable reduction in the eGI when compared to white bread (WB) and control snacks. Incorporating MOLP into snacks causes smoother alterations in postprandial blood glucose, significantly reducing glucose peak, time to peak, and glycemic index (GI). Our findings indicate that MOLP exhibits hypoglycemic potential, offering a scientific foundation for the future development of functional foods aimed at managing diabetes in dogs.

## Simple Summary

*Moringa oleifera* leaf (MOL) and its extracts are recognized for their hypoglycemic properties. The current study assessed the hypoglycemic potential of *Moringa oleifera* leaf peptide (MOLP), emphasizing its inhibitory effects on carbohydrate metabolism-related enzymes in vitro and its influence on postprandial glycemic response in dogs. MOLP markedly inhibited the activities of α-amylase and α-glucosidase, as well as decreased the estimated glycemic index (eGI) of MOLP-containing snacks, thereby lowering the postprandial glycemic response in dogs. These findings indicate that MOLP could serve as a bioactive component in dogs’ foods for the regulation of postprandial glycemia and the management of diabetes.

## 1. Introduction

Pets are regarded as important family members, contributing to social support, companionship, and enhanced mental health for humans [1]. Currently, the population of pets, particularly dogs, and their owning households are increasing rapidly. According to statistical data, over 106.36 million pet dogs are housed in Europe [2], there are 65 million dog-owning households in the US [3], and over 50 million dogs are owned by urban families in China [4]. Nonetheless, alongside improving material conditions, the problem of obesity caused by excess body fat resulting from a long-term imbalance between energy intake and consumption in pets has become an escalating health challenge globally [5]. Survey data from the Association for Pet Obesity Prevention (https://www.petobesityprevention.org/2022, accessed on 31 July 2025) indicate that over 59% of dogs in the U.S. are overweight or obese, a substantial contributor to diabetes and associated complications, ultimately decreasing quality of life. Diet is closely associated with the onset of diabetes, besides genetic and disease factors [6,7]. A long-term high-fat diet intake induces pancreatitis in pets, leading to a decrease in pancreatic β cell viability, which affects insulin synthesis and secretion [8,9]. Also, obesity can trigger insulin resistance in pets, subsequently resulting in diabetes and additional complications, including hypothyroidism, dental disease, kidney disease, and infections [10]. Researches in humans and various animals demonstrate that low-glycemic index (GI) foods can mitigate hyperglycemia, obesity, diabetes, and the risk of cardiovascular disease [11,12,13,14,15]. Therefore, it is theoretically feasible to create low-GI foods to diminish the rate of intestinal starch digestion and postprandial blood glucose levels in pets, thereby mitigating the health issues influenced by persistent hyperglycemia.

*Moringa oleifera* (MO), a species of the *Moringaceae* family, is native to India and is presently planted in numerous tropical regions and countries [16]. MO is a typical medicinal food, with its leaf, root, seed, and bark displaying physiological regulatory activities, such as anti-inflammatory [17], antioxidant [18], antimicrobial [19], and cardioprotective functions [20]. *Moringa oleifera* leaf (MOL) is abundant in proteins, vitamins, minerals, and essential fatty acids, exhibiting exceptional nutritional value and has been used as a nutritional supplement in animal foods [21,22]. Among the many physiological functions, the powerful anti-hyperglycemic efficacy of MOL is noteworthy. Studies have demonstrated that MOL extract significantly inhibits α-glucosidase activity and promotes glucose uptake by adipocytes [23,24]. Numerous animal studies have shown that direct supplementation of MOL powder to diabetic model mice and rats significantly reduces blood glucose concentrations [25,26,27,28]. Moreover, MOL extracts, whether aqueous, methanol, or ethanol based, exhibited the ability to reduce blood glucose levels in diabetic mice, accompanied by elevated antioxidant capacity [29,30,31,32,33,34]. Dietary supplementation with MOL powder was found to increase plasma insulin levels in healthy subjects in a human clinical study [35]. For individuals with diabetes, the addition of MOL powder into their diet for 30 min resulted in a 22.8% reduction in postprandial blood glucose levels, suggesting that MOL has potential as a functional food in the management of hyperglycemia [36]. At present, the hypoglycemic activity of *Moringa oleifera* leaf peptide (MOLP) has not been investigated, despite limited reports revealing that proteins isolated from MO seed or leaf can lower blood glucose in diabetic mice [37]. Consequently, based on the strong evidence of hypoglycemic effects of MOL and its extracts, we hypothesize that MOLP possesses a similar function.

Diet is a cost-effective and health-conscious approach to managing diabetes in pets. Providing snacks is a crucial strategy for comforting and rewarding pets because it provides positive behavioral reinforcement, emotional support, and sensory pleasure, which helps people better bond with their pets. Providing pets with a low GI snack can be beneficial for daily diabetes management. This study evaluated the impact of MOLP on the enzymatic activities associated with carbohydrate metabolism and assessed the estimated glycemic index (eGI) of MOLP-containing snacks and postprandial blood glucose changes in dogs. The findings may support the creation of functional pet foods and snacks that mitigate the risk of diabetes.

## 2. Materials and Methods

The animal use and experimental protocols for this study were reviewed and pre-approved by the Animal Care and Use Committee of South China Agricultural University (permit number: 2025E011).

### 2.1. Preparation of Snacks

The MOLP used in this study was purchased from Guangzhou Benweida Biotechnology Co., Ltd. (Guangzhou, China) The compositional analysis of MOLP is shown in Table 1. To make the experimental snacks, the control snacks were mixed with 3% MOLP. The formulation of the snacks is shown in Table 2.

### 2.2. α-Amylase and α-Glucosidase Activity Inhibition Assay

The enzymes activity inhibition assay was conducted based on the method of Ge et al. with appropriate modifications [38]. Initially, PBS was used to prepare α-amylase (1 U/mL, Coolaber, Beijing, China), α-glucosidase (0.25 U/mL, Macklin Biochemical Technology Co., Ltd., Shanghai, China), and different concentrations of MOLP solutions (0.625, 1.25, 2.5, 5, 10, 20, and 40 mg/mL). The experimental procedures were performed based on the grouping information in Table 3. Briefly, the reagents of each group were added to corresponding EP tubes and incubated at 37 °C for 10 min. Then, a soluble starch solution (50 μL, 1%, Sigma-Aldrich, St. Louis, MO, USA) was added and continued to incubate at 37 °C for 10 min. Subsequently, a DNS solution (100 μL, Solarbio Life Science, Beijing, China) was added to all groups and boiled for 5 min, followed by an ice bath for 3 min. Finally, 1 mL of distilled water was added and mixed thoroughly, and 200 μL of mixed liquids was pipetted into a 96-well plate to detect the absorbance (Abs) at the wavelength of 540 nm. The Acarbose (0.01, 0.05, 0.1, 0.5, 1, 2, and 5 mg/mL, Macklin Biochemical Technology Co., Ltd., Shanghai, China) served as a positive control. The α-amylase activity inhibition rate was calculated using Equation (1):

(1) $$\mathrm{Inhibition}\;\mathrm{rate}\;(\%)\;=\;1-\frac{Abs\;(Sample)\;-\;Abs\;(Sample\;blank)}{Abs\;(Control)\;-\;Abs\;(Blank)}$$

The inhibition assay for α-glucosidase activity was conducted in accordance with the grouping information in Table 4. The reagents of each group were placed into a 96-well plate and incubated at 37 °C for 15 min. Subsequently, *p*-nitrophenyl-α-D-glucopyranoside (PNPG, 50 μL, 5 mM, Sigma-Aldrich, St. Louis, MO, USA) was introduced and incubated at 37 °C for 15 min. Finally, the reaction was terminated by Na_2_CO_3_ (100 μL, 0.2 M) and Abs was detected at 405 nm. The Acarbose (0.2, 0.4, 0.6, 0.8, 1, and 5 μg/mL) served as a positive control. The α-glucosidase activity inhibition rate was calculated using Equation (2):

(2) $$\mathrm{Inhibition}\;\mathrm{rate}\;(\%)\;=\;1-\frac{Abs\;(Sample)\;-\;Abs\;(Sample\;blank)}{Abs\;(Control)\;-\;Abs\;(Blank)}$$

### 2.3. Snack Starch Hydrolysis and eGI Measurement

In vitro digestion of snacks was conducted following the method of Goñi et al. with some modifications [39]. Pepsin (1.6 g, 120 U/g) and HCl-KCl buffer (20 mL, 0.1 M, pH = 1.5) were added to snacks (containing 50 mg starch) with fat and soluble sugars removed, and the mixture was incubated at 37 °C for 30 min, then cooled to room temperature. The pH of digestive fluid was adjusted to 6.9 by NaOH (1 M), after which tryptic amylase (0.2 g, 5000 U/g) and α-glucosidase solution (4 μL, 1 × 10^4^ U/mL) were added and incubated at 37 °C for 0, 30, 60, 90, 120, 150, and 180 min. At each time point, 0.5 mL of thoroughly mixed digestive fluid was aspirated and boiled for 5 min, then centrifuged at 1200× *g* for 10 min using a high-speed centrifuge (DHS LifeScience and Technology Co., Ltd., Beijing, China, NX-1R) to isolate the supernatant. The D-Glucose Content Assay Kit (Beijing Boxbio Science & Technology Co., Ltd., Beijing, China) was utilized to determine the glucose concentration, and the starch hydrolysis rate of snacks was calculated according to Equation (3) [40]. White bread (WB) is usually used as a reference for in vitro studies of GI values (GI value for WB = 100) [41]. The starch hydrolysis curve was plotted with the time point as the horizontal coordinate and the hydrolysis rate as the vertical coordinate. The area under the curve (AUC) of WB, control snacks, and experimental snacks was calculated, with the hydrolysis index (HI) defined as the ratio of the snacks’ AUC to the WB’s AUC. The eGI of the snacks was calculated via Equation (4):

(3) $$\mathrm{Starch}\;\mathrm{hydrolysis}\;\mathrm{rate}\;(\%)\;=\;\frac{Glucose\;content\;(mg)\;\times 0.9}{Total\;starch\;weight\;(mg)}$$

eGI = 39.71 + 0.549 HI(4)

### 2.4. Postprandial Glycemic Response Tests In Vivo

Sixteen healthy adult Beagles (8 males and 8 females) were used for this experiment with an average body weight (BW) of 14.59 ± 0.18 kg and BCS score of 4.13 ± 0.16 [42], aged 3 years. Dogs were fed individually in cages (1.2 m length × 1.2 m width × 1.4 m height) and temperature of the kennel was maintained at 25 °C with 50–60% humidity. The glucose metabolism function was examined using the method of Rankovic et al. with some changes [43]. Briefly, a 0.5 mL of blood sample was collected through a catheter in forelimb cephalic vein of fasting dogs in the morning (labeled as 0 min), after which a 40 mL of glucose solution was administered at the recommended dosage of 2 g/kg BW^0.75^ [44]. Subsequently, blood was collected again at 15, 30, 45, 60, 90, 120, 150, and 180 min after glucose feeding, and blood glucose concentrations were measured twice at each time point using an ACCU-CHEK Performa glucometer (Roche, Basel, Switzerland), ensuring an error of less than 0.3 mmol/L [45]. The experiment was repeated after an interval of two days, and the data from the two measurements were averaged to evaluate whether the glucose metabolism functioned normally.

Prior to the GI test, proximate analyses were conducted on the snacks to determine the amount of available carbohydrates (Av CHO, 50.24% for control snacks and 50.68% for MOLP snacks). Dogs were evenly allocated into a control group (CONT, *n* = 8) and experimental group (MOLP, *n* = 8). A 0.5 mL of blood sample was collected through a catheter in a forelimb cephalic vein of fasting dogs in the morning (labeled as 0 min), and the CONT and MOLP groups were fed 30 g of control and experimental snacks, respectively. Blood was collected again at 15, 30, 45, 60, 90, 120, 150, and 180 min after snacks were fed, and blood glucose concentrations were measured twice at each time point. The blood glucose change curve was plotted with the time points as the horizontal coordinate and the glucose concentrations as the vertical coordinate, and the AUC was calculated. Furthermore, the incremental glucose concentration at each time point was determined relative to the baseline at 0 min, enabling the measurement of the incremental area under the curve (IAUC). The snacks’ GI value was calculated by multiplying the ratio of their IAUC to glucose’ IAUC by 100 [46].

### 2.5. Statistical Analysis

The data are expressed as mean ± standard error of the mean (SEM). All data were verified for normality and homogeneity of variance prior to analysis. The CONT and MOLP groups were compared using the Student’s *t*-test (SPSS, v 28.0). The half-maximal inhibitory concentration (IC_50_) value was computed using GraphPad Prism software (v 10.2.0). The differences were considered significant when *p* < 0.05.

## 3. Results

### 3.1. Enzyme Inhibitory Activity of MOLP In Vitro

**Figure 1:**
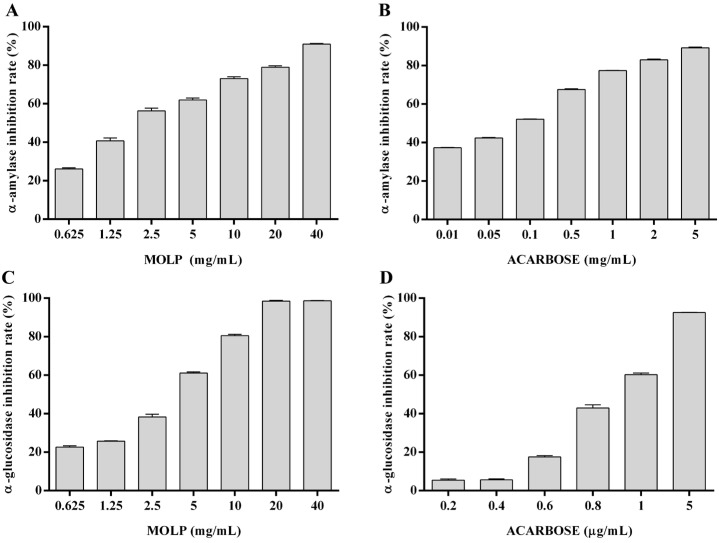
Effects of MOLP and acarbose on enzyme inhibition rate. (**A**,**B**) Inhibition rate of α-amylase by MOLP and acarbose. (**C**,**D**) Inhibition rate of α-glucosidase by MOLP and acarbose.

MOLP exhibited a dose-dependent inhibitory effect on both α-amylase and α-glucosidase activities. MOLP reduced α-amylase activity by 90.98% at a concentration of 40 mg/mL (Figure 1A) and inhibited α-glucosidase by 98.44% at 20 mg/mL (Figure 1C). Acarbose showed the same inhibitory effect (Figure 1B,D). In addition, the IC_50_ of MOLP for α-amylase and α-glucosidase was 2.29 ± 0.10 mg/mL and 2.80 ± 0.04 mg/mL, respectively, both of which exceeded the IC_50_ values of acarbose (Table 5).

### 3.2. MOLP Reduces Snacks Starch Hydrolysis Rate and eGI In Vitro

**Figure 2:**
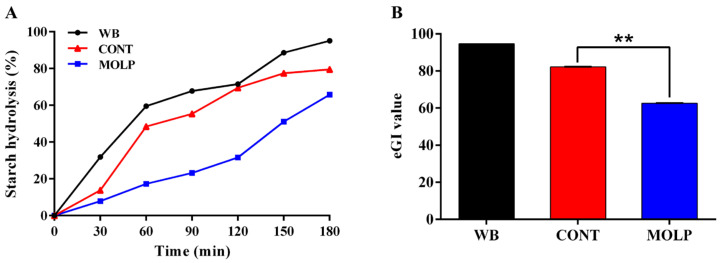
Effects of MOLP on snacks’ starch hydrolysis rate (**A**) and eGI (**B**). “**” indicates *p* < 0.01.

In vitro digestion experiments showed that WB starch was hydrolyzed fastest within 180 min. The starch hydrolysis rate was lower in the MOLP group compared to the CONT group. At 180 min, the starch hydrolysis rate was 79.58% and 65.82% in the CONT and MOLP group, respectively (Figure 2A). In addition, the eGI of the MOLP group was significantly lower than that of the CONT group (*p* < 0.01, Figure 2B).

### 3.3. MOLP Lowers Postprandial Glycemic Response in Dogs

**Figure 3:**
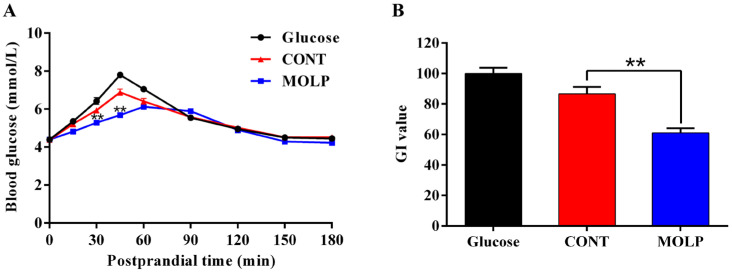
Effects of MOLP on postprandial glucose (**A**) and GI (**B**) of snacks in dogs. “**” indicates *p* < 0.01.

In comparison to the CONT group, dogs in the MOLP group exhibited significantly lower blood glucose levels at 30 and 60 min postprandial (*p* < 0.01, Figure 3A). Moreover, dogs in the MOLP group had a noticeable reduction in postprandial blood glucose peak and a increase in time to peak (*p* < 0.01, Table 6). After calculation, the MOLP group showed notably lower AUC and IAUC of postprandial blood glucose (*p* < 0.01, Table 6), and the GI value of the MOLP-containing snacks was also significantly reduced (*p* < 0.01, Figure 3B).

## 4. Discussion

In the animal digestive system, α-amylase and α-glucosidase serve as essential enzymes to regulate carbohydrate digestion. α-amylase catalyzes the hydrolysis of the starch α-1, 4 glycosidic bond to produce oligosaccharides, which are subsequently processed by α-glucosidase to produce glucose that can be absorbed by intestinal epithelial cells [47]. Studies have indicated that the inhibition of α-amylase and α-glycosidase activities significantly slows down the starch carbohydrate digestibility [48,49,50], thereby serving as an effective strategy for controlling blood glucose and managing type 2 diabetes [51].

Recent studies have validated the enzyme inhibitory activity of MOL and its extracts. Gomes et al. reported that MOL extracts at 1 mg/mL reduced α-amylase and α-glucosidase activities by 79% and 98% [52], respectively, whereas Ferreira observed a 94% activity inhibition rate of α-amylase at the same concentration [53]. It is noteworthy that different extraction methods significantly affected the efficacy of the active ingredient. Magaji discovered a dose-dependent increase in the α-amylase and α-glucosidase inhibition rate by different concentrations of aqueous extracts of MOL with an IC_50_ of 16.3 ± 2.2 mg/mL and 1.5 ± 0.02 mg/mL, respectively. In addition, methanol and ethyl acetate extracts showed comparable inhibitory ability but with differences in IC_50_ [54]. Ademiluyi et al. [55] further revealed the effect of different treatments of MOL on the enzyme activity inhibition, and the IC_50_ of their preparations against α-amylase and α-glucosidase was more than 64 mg/mL and 38 mg/mL, respectively. Our findings suggested that MOLP inhibited α-amylase and α-glucosidase with IC_50_ of 2.29 mg/mL and 2.80 mg/mL, respectively. In the ethanolic extract of MOL, polyphenolic compounds were the major active constituents [52,53]. Studies have shown that plant polyphenols can inhibit the activity of α-amylase and α-glucosidase derived from fungal and animal intestinal [56,57,58], but polyphenols also exhibit different enzyme inhibitory effects due to differences in processing and structure [59,60]. In addition, it was shown that plant-derived peptides with molecular weight below 1000 Da exhibit potent α-amylase and α-glucosidase inhibitory activities [61,62]. The content of peptides with molecular weight lower than 1000 Da in MOLP used in this experiment accounted for 92.05%, suggesting that MOLP has a strong potential for enzyme inhibition. In addition, compared with polyphenols, the small peptides in MOLP may be more likely to be stably bound to the enzyme and thus exert a stronger inhibitory effect due to their smaller molecular weight and higher affinities, which may also be the reason why the inhibitory effect of MOLP is superior to that of MOL extracts or MOL powder, and further validation is needed.

Most existing studies on the hypoglycemic activity of MOL have predominantly focused on rodent models or humans, primarily utilizing crude extracts or whole leaf powder. Jaiswal et al. discovered that diabetic model rats treated with MOL extracts exhibited enhanced glucose tolerance and increased serum insulin levels [29]. Clinical studies indicate that the administration of 50 g of MOL powder led to a 21% reduction in blood glucose levels one hour postprandial in human diabetics [63]. Moreover, the addition of MOL powder into food can decrease blood glucose levels within 180 min postprandially in healthy individuals [64]. At present, there are no studies concerning the active peptide fractions of MOL with respect to pet health. This study employed peptides isolated from MOL to verify their hypoglycemic effect. As we hypothesized, MOLP significantly reduced the starch digestibility and eGI of snacks, as well as postprandial blood glucose levels in dogs, which is beneficial for alleviating pancreatic stress and managing the development of diabetes. Our study is the first to reveal that MOLP may regulate blood glucose via an enzyme inhibitory mechanism, suggesting that MOLP may be another key hypoglycemic active ingredient in MOL. In pets, α-glucosidase inhibitors, such as acarbose, are recommended by the American Animal Hospital Association as therapeutic agents for canine diabetes [65]. Although our results suggest that MOLP is not as effective as acarbose in terms of enzyme inhibition, acarbose may lead to a large amount of undigested sugars entering the colon and fermenting to produce gas, triggering side effects such as bloating and diarrhea [66]. Consequently, MOLP possesses a superior safety profile as a natural ingredient and may be more appropriate for long-term dietary intervention in pets.

The molecular mechanism underlying the enzyme inhibitory function of MOLP may be associated with particular amino acid sequences. Numerous studies indicate that the terminal amino acids of plant peptides that inhibit amylase and glucosidase activities primarily consist of proline, phenylalanine, or valine [67,68,69,70]. We speculate that MOLP also has a similar terminal amino acid composition, but this hypothesis needs to be further verified in the future, and then the binding site of the peptide to the enzymes needs to be clarified by molecular docking techniques. Furthermore, a limitation of this experiment is the unverified efficacy of MOLP in diabetic dogs, which needs to be further explored.

## 5. Conclusions

In summary, MOLP, as a novel moringa leaf-derived active peptide, may effectively regulate the glycemic properties of pet food and canine postprandial blood glucose levels through dual-target enzyme inhibition. This study provides a theoretical basis for the development of pet diabetes management diets based on natural ingredients and suggests a broad application prospect of MOLP in the field of pet nutrition.

## Figures and Tables

**Table 1:** The compositional analysis of MOLP.

Ingredients	Content (%)
**Peptide molecular weight analysis (Dalton, Da)**	
<180	23.84
180–500	53.10
500–1000	15.11
1000–3000	7.28
3000–5000	0.53
5000–10,000	0.11
>10,000	0.04
**Proximate analysis (DM basis, %)**	
Crude protein	47.73
Crude fat	10.01
Crude fiber	5.90
Ash	5.94
Moisture	7.47

**Table 2:** The formulation of snacks.

Ingredients	Proportion (%)	
	Control Snacks	Experimental Snacks
Water	12.10	11.74
Corn flour	9.31	9.03
Potato starch	8.38	8.13
Rice flour	27.93	27.09
Peptone	18.62	18.06
Chicken liver powder	1.86	1.81
Chicken paste	4.65	4.51
Potassium sorbate	0.37	0.36
Chicken meal flour	2.79	2.71
Sodium pyrophosphate	0.47	0.45
Sodium hexametaphosphate	0.47	0.45
Calcium carbonate	1.86	1.81
Glycerol	11.17	10.83
Vitamin E	0.03	0.03
MOLP	-	3.00
**Proximate analysis (DM basis, %)**		
Crude protein	19.84	19.97
Crude fat	2.84	2.71
Crude fiber	0.67	0.74
Ash	7.86	7.63
Moisture	9.05	9.65
Gross energy (kcal/kg)	3874.65	3854.67

**Table 3:** The grouping information of α-amylase activity inhibition assay.

	Sample	Sample Blank	Positive	Control	Blank
MOLP	50 μL	50 μL	-	-	-
α-amylase (1 U/mL)	50 μL	-	50 μL	50 μL	-
PBS	-	50 μL	-	50 μL	100 μL
Acarbose	-	-	50 μL	-	-

**Table 4:** The grouping information of α-glucosidase activity inhibition assay.

	Sample	Sample Blank	Positive	Control	Blank
MOLP	50 μL	50 μL	-	-	-
α-glucosidase (0.25 U/mL)	100 μL	-	100 μL	100 μL	-
PBS	-	100 μL	-	50 μL	150 μL
Acarbose	-	-	50 μL	-	-

**Table 5:** Inhibitory properties of MOLP on enzymes.

Samples	α-Amylase (IC_50_, mg/mL)	α-Glucosidase (IC_50_, mg/mL)
MOLP	2.29 ± 0.10	2.80 ± 0.04
Acarbose	0.06 ± 0.00	1.02 × 10^−3^ ± 0.00

**Table 6:** Changes in dogs’ postprandial blood glucose in the CONT and MOLP groups.

Items	Glucose	CONT	MOLP
Fasted blood glucose (mmol/L)	4.40 ± 0.06	4.38 ± 0.08	4.40 ± 0.05
Peak glucose (mmol/L)	7.83 ± 0.05	7.10 ± 0.06 ^A^	6.24 ± 0.04 ^B^
Time to peak (min)	43.13 ± 1.88	48.75 ± 2.45 ^B^	71.25 ± 5.49 ^A^
AUC (0–180 min)	1002.31 ± 9.87	969.34 ± 7.40 ^A^	924.14 ± 7.46 ^B^
IAUC (0–180 min)	211.70 ± 7.99	183.39 ± 9.85 ^A^	129.36 ± 6.28 ^B^

Note: Different capital letters in the same row represent *p* < 0.01.

## Data Availability

The original contributions presented in this study are included in the article. Further inquiries can be directed to the corresponding author(s).
